# Pneumococcal pneumonia: differences according to blood culture results

**DOI:** 10.1186/1471-2466-14-128

**Published:** 2014-08-05

**Authors:** Alberto Capelastegui, Rafael Zalacain, Amaia Bilbao, Mikel Egurrola, Luis Alberto Ruiz Iturriaga, Jose M Quintana, Ainhoa Gomez, Cristobal Esteban, Pedro P España

**Affiliations:** 1 Service of Pneumology, Hospital de Galdakao-Usansolo, Galdakao, Bizkaia E-48960, Spain; 2 From the Research Unit - Red de Investigación en Servicios de Salud en Enfermedades Crónicas (REDISSEC), Hospital Galdakao-Usansolo, Galdakao, Bizkaia, Spain; 3 From the Pneumology Service, Hospital Universitario Cruces, Barakaldo, Bizkaia, Spain; 4 From the Research Unit, Hospital Universitario Basurto - Red de Investigación en Servicios de Salud en Enfermedades Crónicas (REDISSEC), Bilbao, Bizkaia, Spain

**Keywords:** Pneumococcal pneumonia, Bacteremia

## Abstract

**Background:**

Bacteremia by *Streptococcus pneumoniae* has been traditionally associated with poor outcomes in patients with pneumonia; however, data on its impact on outcomes are limited and are sometimes contradictory.

**Methods:**

We performed a prospective study in two hospitals in northern Spain in which cases diagnosed with pneumococcal pneumonia were selected from a cohort of hospitalized patients with pneumonia between January 2001 and July 2009. We compared patients with pneumococcal bacteremic pneumonia with those with pneumococcal non-bacteremic pneumonia.

**Results:**

We compared 492 patients with negative blood culture and 399 with positive culture results. Host related factors were very similar in both groups. Severity of illness on admission measured by CURB-65 score was similar in both groups. Adjusted analysis showed a greater likelihood of septic shock during in-hospital course among patients with pneumococcal bacteremia (OR, 2.1; 95% CI, 1.2–3.5; P = 0.006). Likewise, patients with positive blood culture had greater in-hospital mortality (OR 2.1; 95% CI, 1.1 - -3.9; P = 0.02), 15-day mortality (OR 3.6; 95% CI, 1.7 - 7.4; P = 0.0006), and 30-day mortality (OR, 2.7; 95% CI, 1.5 - 5; P = 0.002).

**Conclusions:**

Although host related factors and severity on admission were very similar in the two groups, bacteremic patients had worse in-hospital course and outcomes. Bacteraemia in pneumococcal pneumonia is of prognostic significance.

## Background

Despite the introduction of pneumococcal vaccination and advances in antimicrobial agents, case-fatality rates among adults with bacteremic pneumococcal pneumonia vary significantly (ranging from 6% to 30%); they have improved little in the past three decades and, in general, remain high [1-6]. In addition, bacteremic pneumococcal pneumonia continues to evolve, and regular comprehensive analysis of this entity is necessary.

The severity of sepsis can be graded, using the American Collage of Chest Physicians/Society of Critical Care Medicine classification [7], into different progressive stages: bacteremia, systemic inflammatory response syndrome (SIRS), sepsis, severe sepsis, septic shock, and multiple organ dysfunctions. Although there is a hierarchical continuum of severity across sepsis, severe sepsis, septic shock, and multiple organ dysfunction [8], the presence of SIRS has no prognostic significance [9,10], and the prognostic significance of bacteremia remains unclear. Among patients with pneumonia, bacteremia due to *Streptococcus pneumoniae* has traditionally been associated with poor outcomes, it being considered an invasive form of infection. To date, however, there has been little research on the impact of *Streptococcus pneumoniae* bacteremia on the outcome of pneumococcal pneumonia: most studies have focused on bacteremic infection [5,11-13], or on the impact of antibiotic resistance on clinical outcome [14-16], few reports having compared the clinical outcomes of pneumonia patients with and without pneumococcal bacteremia. Moreover, among the few existing comparative studies the findings are contradictory and characteristics of some of the studies have limited their generalizability: the enrolment of relatively small numbers of patients [17-20]; collection of information from a single institution [21,22]; and no adjusted analysis [17,23,24].

Our main objective was to assess whether bacteremia in patients with pneumonia was related to severity on admission, septic shock at admission or during hospitalization, and mortality in a large pneumococcal pneumonia study. We hypothesized that the presence of bacteremia would be associated with higher severity on admission, and also higher rates of shock and mortality due to a greater degree of systemic invasion.

## Methods

### Study population, design and setting

We analysed 4389 adult (18 years or older) patients hospitalized with pneumonia between January 2001 and July 2009. For this study, we selected patients diagnosed with pneumococcal pneumonia and compared the subgroups in this sample with bacteremic and non-bacteremic pneumonia. All patients with a diagnosis of pneumonia and at least one positive blood culture for *Streptococcus pneumoniae* taken within 48 hours of presentation to the hospital were included in the “pneumococcal bacteremic” group. The “pneumococcal non-bacteremic” group included patients with positive *Streptococcus pneumoniae* antigen in urine and negative blood cultures . Any individuals with concurrent meningitis and/or endocarditis were excluded from the analysis.

Data were collected prospectively from two hospitals (Galdakao-Usansolo Hospital and Cruces University Hospital) in the Basque Country (northern Spain). Galdakao-Usansolo Hospital is a 400-bed general teaching hospital serving a population of 300,000, while Cruces University Hospital is a nearby large teaching hospital with a catchment population of 400,000.

Patients were treated empirically with antibiotics according to local practice guidelines: betalactam in combination with macrolides, levofloxacin or betalactamics. Medical care following discharge was determined by patient's health-care providers. No interventions were instigated as part of this study.

### Study variables

All patients’ clinical and demographic characteristics were recorded, as well as their vaccination status and any previous antibiotic treatment for the current episode. To measure the severity of pneumonia upon admission to the emergency department, we used the CURB-65 (Confusion, Urea nitrogen, Respiratory rate, Blood pressure, age ≥65 years) score [25].

Process-of-care variables included: 1) whether appropriate antibiotics were given (defined as an initial antibiotic treatment consistent with the recommendations of Spanish Thoracic Society [SEPAR] [26]: third generation cephalosporins or amoxicillin-clavulanic acid plus a macrolide, or levofloxacin in monotherapy for patients admitted to a hospital ward; non-antipseudomonal cephalosporin plus a macrolide, or levofloxacin instead of macrolide for patients admitted to an intensive care unit)); and 2) and 3) whether antibiotics were administered within 4 or within 8 hours of arrival at the emergency department, respectively; as well as 4) length of antibiotic therapy; 5) length of intravenous antibiotic therapy; and 6) the type of antibiotics given.

Clinical in-hospital measures included: whether the patient 1) was admitted to the intensive care unit (ICU); 2) received mechanical ventilation; or 3) developed septic shock; as well as whether there was 4) treatment failure; or 5) severe sepsis.

Outcome measures included: 1) in-hospital mortality; 2) and 3) mortality at 15 and 30 days after admission; 4) hospital readmission within 30 days; and 5) length of hospital stay (calculated as the date of discharge minus the date of admission).

This study was approved by Galdakao Ethics Committee and Cruces University Hospital Ethics Committee.

The formal consent to participate was verbal because this study was not interventional.

### Definitions

Pneumonia was defined as pulmonary infiltrate on a chest X-ray not known to have pre-existed and symptoms consistent with pneumonia, including cough, dyspnoea, fever, and/or pleuritic chest pain. Patients with pneumonia were excluded if they were known to be positive for human immunodeficiency virus, were chronically immunosuppressed (defined as immunosuppression for solid organ transplantation, postsplenectomy, receiving ≥10 mg/day of prednisone or the equivalent for more than 30 days, treatment with other immunosuppressive agents, or neutropenia, i.e., <1.0 × 10^9^/L neutrophils), had been hospitalized for the previous 14 days before the diagnosis of pneumonia, or had hospital-acquired pneumonia.

Septic shock was defined as systolic blood pressure <90 mmHg and the need for vasopressors for 4 hours or more, while severe sepsis was defined as sepsis associated with organ dysfunction and perfusion abnormalities [27]. Treatment failure was defined as clinical deterioration during hospitalization with hemodynamic instability, confirmation of respiratory failure or the onset thereof, the institution of mechanical ventilation, demonstrated radiological progression of pneumonia or the appearance of a new focus of infection, or persistent fever or the reappearance of fever if a change in treatment was needed [28].

### Bacteriological studies

The strategy for pneumococcal diagnosis included blood cultures and a urinary antigen test during the first 24 hours after arrival at hospital. The detection of *Streptococcus pneumoniae* was performed by analysing concentrated urine samples with an immunochromatographic membrane assay (Binax Inc; Scarborough, ME). An etiologic diagnosis of pneumococcal pneumonia was considered to be definitive if one or both of the following criteria were met: 1) isolation of *Streptococcus pneumoniae* in a sterile specimen (blood and pleural fluid); and/or 2) positive urinary antigen test for *Streptococcus pneumoniae*.

### Statistical analysis

Descriptive statistics included frequency tables and mean and standard deviation (SD). Patient characteristics, process of care, in-hospital course and outcomes were compared stratifying by blood culture result (positive vs. negative). Chi-square and Fisher’s exact tests were performed for the comparison of categorical variables, and the Student’s *t*-test or nonparametric Wilcoxon tests were performed for continuous variables.

Univariate logistic regression models were also used to compare in-hospital course and outcomes between the two groups of patients (unadjusted results). Then, multivariate logistic regression models were built for the comparison, adjusting for severity of illness at admission, measured by CURB-65, as well as for patient characteristics and variables related to the process of care found to be significantly different in the groups stratified by blood culture results. In the final multivariate models, only adjusting variables found to be statistically significant were kept. We determined odds ratios (ORs) and 95% confidence intervals (95% CIs). For comparing lengths of hospital stay, a general linear model was built, and due to the skewed distribution of length of stay, the logarithmic transformation was used.

Finally, Kaplan-Meier curves were constructed for 15- and 30-day mortality for each group of patients, and comparisons were performed with the log-rank test. Further, Cox proportional hazards model was used to compare survival between the two groups of patients adjusting for the same variables as stated previously. We determined the hazard ratios (HRs) and 95% CIs.

All effects were considered significant at P < 0.05. All statistical analysis was performed using SAS for Windows, version 9.2 (SAS Institute, Cary, NC) and S-Plus 2000 (MathSoft Inc., 1999).

## Results

A total of 891 patients were identified in the study period with a diagnosis of pneumococcal pneumonia and with blood culture results. Pneumococcal bacteremia was identified in 399 (44.8%) cases. The group of pneumococcal non-bacteremic pneumonia included 492 (55.2%) cases, all of them with positive antigen in urine and negative blood culture. The patient characteristics are summarized in Table 1 by the blood culture result. Host-related factors were very similar in the two groups, although statistically significant differences were found in sex, alcoholism, pneumococcal vaccine, congestive heart failure, blood urea nitrogen and the radiological findings on admission. Patients with positive blood cultures had higher rates of bilateral or multilobe radiological involvement and pleural effusion and were less likely to have had the pneumococcal vaccine in the last 5 years. Severity of illness on admission measured by the CURB-65 score was similar in the two groups. A total of 395 (99.9%) of 399 blood isolates were available for in vitro susceptibility testing. In nine (2.3%) cases, pneumococci was highly resistant to penicillin (minimum inhibitory concentration ≥ 4 μg/ml).

**Table 1:** **Characteristics of patients hospitalized with pneumonia by *Streptococcus pneumoniae *** **by blood culture result**

**Characteristics**	**Blood culture positive (N = 399)**	**Blood culture negative (N = 492)**	***P*** **value**
Age, years, mean (SD)	63.6 (18.5)	65.2 (17)	0.2
Age ≥65 years	225 (56.4)	290 (58.9)	0.4
Age >75 years	130 (32.6)	167 (33.9)	0.7
Women	131 (32.8)	210 (42.7)	0.003
Underlying diseases			
Cancer	27 (6.8)	17 (3.5)	0.2
Liver disease	18 (4.5)	12 (2.4)	0.1
Congestive heart failure	54 (13.5)	43 (8.7)	0.02
Cerebrovascular disease	22 (5.5)	32 (6.5)	0.5
Renal disease	27 (6.8)	24 (4.9)	0.2
Chronic obstructive pulmonary disease	74 (18.6)	116 (23.6)	0.07
Diabetes mellitus	60 (15.1)	97 (19.8)	0.07
Number of comorbid conditions			0.96
0	203 (50.9)	253 (51.4)	
1	130 (32.6)	156 (31.7)	
≥2	66 (16.5)	83 (16.9)	
Nursing home resident	13 (3.3)	20 (4.1)	0.5
Smoking			0.06
No	130 (43.5)	225 (47.8)	
Yes	86 (28.8)	100 (21.2)	
Ex-smoker	83 (27.8)	146 (31)	
Alcoholism	58 (15.3)	44 (9.4)	0.008
Influenza vaccine in the last year	93 (26.4)	149 (30.9)	0.2
Pneumococcal vaccine in the last 5 years	14 (3.8)	121 (25.5)	<0.0001
**Findings on physical examination on admission**			
Altered mental status	39 (9.8)	49 (10)	0.9
Pulse ≥ 125/min	62 (15.6)	62 (12.6)	0.2
Respiratory rate ≥ 30/min	98 (24.8)	96 (19.5)	0.06
Systolic blood pressure < 90 mmHg	30 (7.5)	42 (8.5)	0.6
Temperature < 35°C or ≥ 40°C	7 (1.8)	4 (0.8)	0.2
**Laboratory findings on admission**			
Blood urea nitrogen > 30 mg/dL	192 (48.1)	163 (33.1)	<0.0001
Glucose ≥ 250 mg/dL	39 (9.8)	38 (7.7)	0.3
Hematocrit < 30%	10 (2.5)	23 (4.7)	0.1
Sodium < 130 mmol/L	33 (8.3)	22 (4.5)	0.02
PaO_2_ < 60 mmHg	188 (47.1)	209 (42.5)	0.2
Arterial pH < 7.35	31 (7.8)	24 (4.9)	0.07
**Radiological findings on admission**			
Bilateral or multilobe radiological involvement	142 (35.7)	122 (24.8)	0.0004
Pleural effusion	65 (16.3)	43 (8.7)	0.0006
**Severity of illness on admission**			
CURB65 score^*^			0.07
0,1	145 (36.3)	215 (43.7)	
2	159 (39.9)	167 (33.9)	
>2	95 (23.8)	110 (22.4)	

SD, standard deviation.

Data are expressed as numbers (percentage) unless otherwise stated. Percentages exclude patients with missing data.

^*^Severity of illness on admission assessed with CURB-65 (Confusion, Urea nitrogen, Respiratory rate, Blood pressure, age ≥65 years) score.

Process of care indicators in both groups are shown in Table 2. Statistically significant differences were observed in antibiotic management between the two groups. In particular, the use of antibiotics was appropriate according to SEPAR guidelines in 85.3% of patients with negative cultures and just 68.6% of those with positive cultures. In both groups, however, over 90% of patients received antibiotics within 8 hours and the length of antibiotic therapy was similar.

**Table 2:** **Process-of-care of patients hospitalized with pneumonia by** ***Streptococcus pneumoniae*** **by blood culture result**

**Process-of-care**	**Blood culture positive (N = 399)**	**Blood culture negative (N = 492)**	***P *** **value**
Previous antibiotic treatment	26 (6.5)	56 (11.4)	0.013
Appropriate antibiotic^*^	273 (68.6)	419 (85.3)	<0.0001
Antibiotics within 4 hours	257 (73)	389 (79.7)	0.019
Antibiotics within 8 hours	330 (93.8)	471 (96.5)	0.06
Length of antibiotic therapy, days, mean (SD)^†^	14.7 (7.2)	13.9 (4.6)	0.9
Length of intravenous antibiotic therapy, days, mean (SD)^†^	7 (7.2)	5.9 (4.8)	0.4
Antibiotic treatment			<0.0001
Beta-lactam monotherapy	93 (23.4)	113 (23)	
Beta-lactam/macrolide	13 (3.3)	30 (6.1)	
Fluoroquinolones	257 (64.6)	346 (70.5)	
Macrolide monotherapy	1 (0.3)	0 (0)	
Others	34 (8.5)	2 (0.4)	
Dual antibiotic therapy including a macrolide	13 (3.3)	31 (6.3)	0.037

SD, standard deviation.

Data are given as number (percentage) unless otherwise indicated. The percentage excluded patients with missing data.

^*^Appropriate antibiotic defined as usage of antibiotics recommended in the guidelines of the SEPAR.

^†^Deaths are excluded.

In-hospital course and outcome indicators in the two groups are shown in Table 3 (unadjusted analysis). Patients with pneumococcal bacteremia had significantly higher rates of mechanical ventilation use, septic shock and treatment failure during the hospitalization, and higher in-hospital, 15-day and 30-day mortality, as well as longer hospital stays.

**Table 3:** **In-hospital course and outcomes of patients hospitalized with pneumonia by *Streptococcus pneumoniae *** **by blood culture result**

**In-hospital evolution and outcomes**	**Positive blood culture (N = 399)**	**Negative blood culture (N = 492)**	***P*** **value**	**Odds ratio (95% CI)**
**In-hospital course**				
Admission to intensive care unit	92 (23.1)	101 (20.5)	0.4	1.2 (0.8 – 1.6)
Need for mechanical ventilation	42 (10.5)	27 (5.5)	0.005	2 (1.2 – 3.4)
Septic shock^*^	53 (14.9)	33 (6.7)	0.0001	2.4 (1.5 – 3.8)
Treatment failure	72 (18.2)	59 (12.1)	0.01	1.6 (1.1 – 2.3)
Severe sepsis	185 (46.4)	203 (41.3)	0.1	1.2 (0.9 – 1.6)
**Outcomes**				
In-hospital mortality	35 (8.8)	22 (4.5)	0.009	2.1 (1.2 – 3.6)
15-day mortality	33 (8.3)	14 (2.9)	0.0003	3.1 (1.6 – 5.8)
30-day mortality	37 (9.3)	18 (3.7)	0.0005	2.7 (1.5 – 4.8)
30-day readmission	10 (3.3)	27 (5.6)	0.1	0.6 (0.3 – 1.2)
Length of hospital stay (days)^†^				
Mean (SD)	10 (13.7)	7 (5.5)	0.02	1.2 (1.04 – 1.3)^║^
Median (IRQ)	6 (4 – 10)	6 (4 – 8)	0.02	
>3 days	295 (81)	373 (79.4)	0.5	1.1 (0.8 – 1.6)

SD, standard deviation; CI, Confidence interval; IRQ, Interquartile range.

Data are given as number (percentage) unless otherwise indicated. Percentages exclude patients with missing data. Treatment failure is defined in the text.

^*^Septic shock is defined as arterial systolic blood pressure <90 mm Hg and the need for vasopressors for a minimum of 4 hours.

^**†**^Deaths are excluded.

^║^For the comparison of length of hospital stay as a continuous variable, the general linear model was used, and due to the skewed distribution of length of stay, the logarithmic transformation was applied. Hence, data are given as the exponential of the estimated beta parameter, indicating how many times longer the length of stay was among blood culture-positive than culture-negative patients with *Streptococcus pneumoniae*.

**Figure 1:**
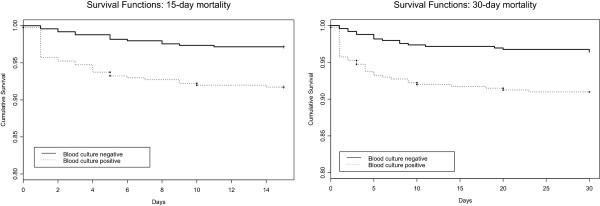
Kaplan-Meier survival curves for the blood culture positive and negative patients.

Table 4 shows the comparison of in-hospital course and outcomes in the two groups adjusting for severity of illness at admission, measured by CURB-65, as well as for patient characteristics and variables related to the process of care found to be significantly different in the two groups of patients, such as sex, congestive heart failure, alcoholism, pneumococcal vaccine in last 5 years, pleural effusion, appropriate antibiotic, antibiotics within 4 hours, dual antibiotic therapy including a macrolide, and antibiotic administration prior to hospital admission. A higher likelihood of septic shock (OR, 2.1; 95% CI, 1.2 – 3.5; P = 0.006) during the hospital stay was found among patients with pneumococcal bacteremia. Likewise, patients with positive blood cultures had higher in-hospital mortality (OR 2.1; 95% CI, 1.1 - 3.9; P = 0.02), 15-day mortality (OR, 3.6; 95%CI, 1.7 - 7.4; P = 0.0006), and 30-day mortality (OR, 2.7; 95% CI, 1.5 - 5; P = 0.002). Kaplan-Meier survival curves for each of the groups demonstrate markedly different survival trajectories in 15- and 30-day mortality (Figure 1). Adjusted cox proportional hazards models confirmed the previous comparison of survival between the two groups of patients: for 15-day mortality, the HR was 3.3 (95% CI, 1.7 - 6.5, P = 0.0005); and for 30-day mortality, the HR was 2.8 (95% CI, 1.6 - 5.1, P = 0.0006).

**Table 4:** **Comparison between in-hospital course and outcomes of patients hospitalized with pneumonia by *Streptococcus pneumoniae *** **according the blood culture results: adjusted analysis**

	Odds ratio (95% CI)*	***P *** **Value**
**In-hospital course**		
Admission to intensive care unit	1 (0.7 – 1.4)	0.8
Use of mechanical ventilation	1.7 (1 – 3.1)	0.06
Septic shock^†^	2.1 (1.2 – 3.5)	0.006
Treatment failure	1.4 (1 – 2.1)	0.06
Severe sepsis	1.1 (0.8 – 1.5)	0.4
**Outcomes**		
In-hospital mortality	2.1 (1.1 – 3.9)	0.02
15-day mortality	3.6 (1.7 – 7.4)	0.0006
30-day mortality	2.7 (1.5 – 5)	0.002
30-day readmission	0.5 (0.2 – 1.1)	0.08
Length of hospital stay (days)^**‡**^		
Continuous	1.1 (1 – 1.2)^║^	0.1
>3 days	1 (0.7 – 1.4)	0.8

CI, confidence interval.

^*^Odds ratio adjusted by severity of illness on admission measured by CURB-65, and those characteristics of patients and variables related to process-of-care which were found statistically significant according to blood culture results, such as sex, congestive heart failure, alcoholism, pneumococcal vaccine in last 5 years, pleural effusion, appropriate antibiotic, antibiotics within 4 hours, dual antibiotic therapy including a macrolide, and antibiotic administration prior to hospital admission. Only significant adjusting variables were kept in each model.

^†^Septic shock defined as arterial systolic blood pressure <90 mm Hg and need for vasopressors ≥ 4 hours.

^‡^Deaths are excluded.

^║^For the comparison of length of hospital stay as a continuous variable, the general linear model was used, and due to the skewed distribution of length of stay, the logarithmic transformation was applied. Hence, data are given as the exponential of the estimated beta parameter, indicating how many times longer the length of stay was among blood culture-positive than culture-negative patients with *Streptococcus pneumoniae*.

## Discussion

Our findings confirm that there are substantial differences in in-hospital course and outcomes among patients hospitalized with pneumonia due to *Streptococcus pneumoniae* as a function of their blood culture results. We found that patients with pneumococcal bacteremia have a poorer in-hospital course – in terms of septic shock – and poorer outcomes – in terms of in-hospital, 15- and 30-day mortality. Notably, we also identified that the illness severity on admission assessed by CURB-65 score was similar in the two groups.

Our study is important as comparing bacteremic with non-bacteremic pneumoccocal pneumonia we have identified that both course and outcomes are poorer for bacteremic patients while they show a similar severity of illness on admission. There is one previous study with the same design and similar results; the differences observed in the mortality were not, however, adjusted for host-related factors or antibiotic treatment [18]. Others strengths of this study are its prospective design, identification of cases based on clinical diagnosis, relatively large sample of non-selected patients, comprehensive assessment of outcomes, detailed collection of clinical data, and use of a robust risk-adjustment model.

When comparing characteristics of two groups of patients, the similarities rather than the differences are initially what are most important. In our study, patients were similar in age, level of comorbidity, and severity of illness on admission. Observed gender differences are consistent with the results of other studies [29,30], suggesting that women are less likely to develop sepsis, maybe related to the sex hormones or anatomic, lifestyle and behavioural differences [31]. Blood urea nitrogen, higher in bacteremic patients group, is an independent variable associated with the severity of pneumonia [25]. The dehydration, which is common in older patients hospitalized for pneumonia [32], may also contribute to a higher urea level. Besides, the rate of bilateral or multilobe radiological involvement and pleural effusion were significantly higher in bacteremic patients. Our findings indicate that the bacteremic patients had a poorer prognosis and higher case-fatality rate, while the illness severity on admission was similar in the two groups as assessed by the CURB-65 score. It is possible that bacteremic pneumococcal pneumonia adds some features that are not captured by this severity score.

The fact that clinical outcomes of pneumonia patients are different depending on whether or not they have pneumococcal bacteremia is an important long-standing issue that has yet to be fully understood. Our study adds new data on this issue in that it shows that the bacteremia is associated with poorer in-hospital course and outcomes. There are previous studies [17-20] with contradictory findings, although all of these have a small sample size without the adequate power to detect outcome differences between bacteremic and non-bacteremic groups of patients.

In agreement with our findings, several studies [14,21,22,24] have found that bacteremia is a risk factor for death in patients with pneumonia. A meta-analysis [24] identified 11 factors, including bacteremia, with statistically significant associations with mortality in patients with pneumonia; however, the authors were unable to determine whether these factors are independently associated with mortality due to the nature of the primary data. Garcia-Vidal et al. [21], in a study carried out in a single hospital, identified pneumoccocal bacteremia as an independent factor associated with early death in patients with pneumonia. Further, a recent study [22] performed in one hospital in Taiwan found that the presence of *Streptococcus pneumoniae* bacteremia predicted mortality in pneumococcal pneumonia, although these authors included immunosuppressed patients and children. In contrast to our study, however, a Canadian multicentre study [23] showed similar outcomes in bacteremic pneumococcal pneumonia and non-bacteremic pneumonia, though using non-adjusted analysis. The low mortality rate (of 5.3%) among patients with pneumococcal bacteremia in that study is attributable to the fact that the most severely ill patients were often not enrolled. An international, retrospective study [33] concluded that pneumococcal bacteremia does not increase the risk of poor outcomes in patients with pneumonia; ICU admission rate and the non-adjusted pneumonia-mortality were, however, significantly higher in the pneumococcal bacteremic pneumonia group. In a Spanish multicentre study [34] conducted in patients with pneumonia admitted to the ICU, bacteremia was not found to affect outcomes: in this case, the results may be due in part to enrolment bias, because the requirements for ICU admission in Spain have a selective approach for patients with advanced age and chronic risk factors.

Although we have assessed the differences in mortality between bacteremic and non-bacteremic groups adjusting for the antibiotic treatment used, the prescription of an antibiotic was appropriate according SEPAR guidelines in less than 70% of cases in the bacteremic group, because of the use of beta-lactam antibiotics alone. This treatment may be considered suboptimal because previous research [35,36] suggests a benefit of combination therapies, including a macrolide, applied to pneumonia associated with *Streptococcus pneumoniae* bacteremia. On the other hand, these studies are hampered by design limitations, and their conclusions should be interpreted with caution [37].

A potential weakness should be noted. In the current study, the ratio of bacteremic to non-bacteremic episodes was 81.1% (399/492 patients) when the percentage of patients with pneumococcal pneumonia and positive blood culture does not usually exceed 30%. In this study, only cases with blood culture results were included. The number of diagnosed cases of pneumococcal pneumonia was higher during the study period. This was due to the fact that requests for blood cultures in pneumonia are optional and depend on the judgment of the attending physician. In fact, it is accepted clinical practice not to request a blood culture once an immediate diagnosis has been obtained by the urinary antigen test. An another question to take into account. Inclusion criteria for “pneumococcal non-bacteremic” group were stringent in order to achieve an unquestionable diagnosis. Only the patients with positive antigen in urine and negative blood culture were included in this group. For this study we excluded patients whose diagnosis was just based on sputum culture, because a diagnosis method of pneumococcal pneumonia that does not depend on sputum culture is desirable.

## Conclusions

We have examined the differences in pneumococcal pneumonia as a function of blood culture results. Although the host-related factors and severity on admission were very similar in the two groups, bacteremic patients had a poorer in-hospital course and outcomes. Bacteremia in pneumococcal pneumonia has prognostic significance given that is associated with poorer outcomes.

## Abbreviations

CI: Confidence interval; CURB 65: Confusion, Urea nitrogen, Respiratory rate, Blood pressure, age ≥65 years; HR: Hazard ratio; ICU: Intensive care unit; OR: Odds ratio; SIRS: Systemic inflammatory response syndrome; SEPAR: Spanish Thoracic Society.

## Competing interest

The authors have no competing interest to declare and sponsors had no role in this study.

## Authors’ contributions

AC, RZ, AB, LARI, JMQ, and PPE conceived and designed the study. RZ, ME, LARI, AG, and CE enrolled patients and collected and compiled data. AB performed the statistical analysis. AC, RZ, AB, ME AG, JMQ, and PPE analyzed and interpreted the data. AC, RZ, and AB wrote the manuscript. ME, LARI, AG CE, JMQ, and PPE commented and revised the report. All authors read and approved the final manuscript.

## Pre-publication history

The pre-publication history for this paper can be accessed here:

http://www.biomedcentral.com/1471-2466/14/128/prepub

## References

[B1] Berjohn CM Fishman NO Joffe MM Edelstein PH Metalay JP Treatment and outcomes for patients with bacteremic pneumococcal pneumonia Medicine 2008 87 160 166 18520325 10.1097/MD.0b013e318178923a

[B2] Kalin M Ortqvist A Almela M Kalin M Ortqvist A Almela M Aufwerber E Dwyer R Henriques B Jorup C Julander I Marrie TJ Mufson MA Riquelme R Thalme A Torres A Woodhead MA Prospective study of prognostic factors in Community-acquired bacteremic pneumococcal disease in 5 countries J Infect Dis 2000 182 840 847 10950779 10.1086/315760

[B3] Mufson MA Stanek RJ Bacteremic pneumococcal pneumonia in one American city: a 20-year longitudinal study, 1978-1997 Am J Med 1999 107 Suppl 34S 43S 10451007 10.1016/s0002-9343(99)00098-4

[B4] Garcia-Vidal C Ardanuy C Tubau F Garcia-Vidal C Ardanuy C Tubau F Viasus D Dorca J Liñares J Gudiol F Carratalà J Pneumococcal pneumonia presenting with septic shock: host- and pathogen-related factors and outcomes Thorax 2010 65 77 81 19996337 10.1136/thx.2009.123612

[B5] Watanakunakom C Bailey TA Adult bacteremic pneumococcal pneumonia in a community teaching hospital…. 1992–1996: a detailed analysis of 108 cases Arch Intern Med 1997 157 1965 1971 9308508

[B6] Plouffe JF Breiman RF Facklam RR Bacteremia with *Streptococcus pneumoniae*: implications for therapy and prevention JAMA 1996 275 194 198 8604171 10.1001/jama.275.3.194

[B7] Bone RC Balk RA Cerra FB Dellinger RP Fein AM Knaus WA Schein RM Sibbald WJ ACCP/SCCM Consensus Conference Committee Definitions for sepsis and organ failure and guidelines for the use of innovative therapies in sepsis. The ACCP/SCCM Consensus Conference Committee Chest 1992 101 1644 1655 1303622 10.1378/chest.101.6.1644

[B8] Alberti C Brun-Buisson C Chevret S Antonelli M Goodman SV Martin C Moreno R Ochagavia AR Palazzo M Werdan K Le Gall JR European Sepsis Study Group Systemic inflammatory response and progression to severe sepsis in critically ill infected patients Am J Respir Crit Care Med 2005 171 461 468 15531752 10.1164/rccm.200403-324OC

[B9] Alberti C Brun-Buisson C Goodman SV Guidici D Granton J Moreno R Smithies M Thomas O Artigas A Le Gall JR European Sepsis Group Influence of systemic inflammatory response syndrome and sepsis on outcome of critically ill infected patients Am J Respir Crit Care Med 2003 168 77 84 12702548 10.1164/rccm.200208-785OC

[B10] Dremsizov T Clermont G Kellum JA Kalassian KG Fine MJ Angus DC Severe sepsis in community-acquired pneumonia. When does it happen, and do systemic inflammatory response syndrome criteria help predict course? Chest 2006 129 968 978 16608946 10.1378/chest.129.4.968

[B11] Watanakunakorn C Greifenstein A Stroh K Jarjoura DG Blend D Cugino A Ognibene AJ Pneumococcal bacteremia in three community teaching hospitals from 1980 to 1989 Chest 1993 103 1152 1156 8131456 10.1378/chest.103.4.1152

[B12] Torres JM Cardenas O Vasquez A Schlossberg D Streptococcus penumoniae bacteremia in a community hospital Chest 1998 113 387 390 9498956 10.1378/chest.113.2.387

[B13] Mufson MA Kruss DM Wasil RE Metzger WI Capsular types and outcome of bacteremic pneumococcal disease in the antibiotic era Arch Intern Med 1974 134 505 510 4152800

[B14] Song JH Jung SI Ki HK Shin MH Ko KS Son JS Chang HH Kim SW Lee H Kim YS Oh WS Peck KR Chongthaleong A Lalitha MK Perera J Yee TT Jamal F Kamarulzaman A Carlos CC So T Asian Network for Surveillance of Resistant Pathogens Study Group Clinical outcomes of pneumococcal pneumonia caused by antibiotic-resistant strains in Asian countries: a study by the Asian network for surveillance of resistant pathogens Clin Infect Dis 2004 38 1570 1578 15156445 10.1086/420821

[B15] Yu VL Chiou CC Feldman C Ortqvist A Rello J Morris AJ Baddour LM Luna CM Snydman DR Ip M Ko WC Chedid MB Andremont A Klugman KP International Pneumococcal Study Group An international prospective study of pneumococcal bacteremia: correlation with in vitro resistance, antibiotics administered, and clinical outcome Clin Infect Dis 2003 37 230 237 12856216 10.1086/377534

[B16] Moroney JF Fiore AE Harrison LH Patterson JE Farley MM Jorgensen JH Phelan M Facklam RR Cetron MS Breiman RF Kolczak M Schuchat A Clinical outcomes of bacteremic pneumococcal pneumonia in the era of antibiotic resistance Clin Infect Dis 2001 33 797 805 11512085 10.1086/322623

[B17] Bohte R van Furth R van den Broek P Aetiology of community-acquired pneumonia: a prospective study among adults requiring admission to hospital Thorax 1995 50 543 547 7597669 10.1136/thx.50.5.543 PMC1021226

[B18] Musher DM Alexandraki I Graviss EA Yanbeiy N Eid A Inderias LA Phan HM Solomon E Bacteremic and nonbacteremic pneumococcal pneumonia: a prospective study Medicine 2000 79 210 221 10941350 10.1097/00005792-200007000-00002

[B19] Brandenburg JA Marrie TJ Coley CM Singer DE Obrosky DS Kapoor WN Fine MJ Clinical presentation, processes and outcomes of care for patients with pneumococcal pneumonia J Gen Intern Med 2000 15 638 646 11029678 10.1046/j.1525-1497.2000.04429.x PMC1495594

[B20] Jover F Cuadrado JM Andreu L Martínez S Cañizares R de la Tabla VO Martin C Roig P Merino J A comparative study of bacteremic and non-bacteremic pneumococcal pneumonia Eur J Intern Med 2008 19 15 21 18206596 10.1016/j.ejim.2007.03.015

[B21] Garcia-Vidal C Fernández-Sabé N Carratalà J Díaz V Verdaguer R Dorca J Manresa F Gudiol F Early mortality in patients with community-acquired pneumonia: causes and risk factors Eur Respir J 2008 32 733 739 18508820 10.1183/09031936.00128107

[B22] Lin SH Lai CC Tan CK Liao WH Hsueh PR Outcomes of hospitalized patients with bacteraemic and non-bacteraemic community-acquired pneumonia caused by Streptococcus pneumoniae Epidemiol Infect 2011 139 1307 1316 20974020 10.1017/S0950268810002402

[B23] Marrie TJ Low DE De Carolis E and the Canadian Community-Acquired Pneumonia Investigators A comparison of bacteremic pneumococcal pneumonia with nonbacteremic community-acquired pneumonia of any etiology –Results from a Canadian multicentre study Can Respir J 2003 7 368 374 14571288 10.1155/2003/862856

[B24] Fine MJ Smith MA Carson CA Mutha SS Sankey SS Weissfeld LA Kapoor WN Prognosis and outcomes of patients with community-acquired pneumonia: a metaanalysis JAMA 1996 275 2 134 141 8531309

[B25] Lim WS van der Eerden MM Laing R Boersma WG Karalus N Town GI Lewis SA Macfarlane JT Defining community-acquired pneumonia severity on presentation to hospital: an international derivation and validation study Thorax 2003 58 377 382 12728155 10.1136/thorax.58.5.377 PMC1746657

[B26] Menéndez R Torres A Aspa J Capelastegui A Prat C Rodríguez de Castro F Sociedad Española de Neumología y Cirugía Torácica Community-acquired pneumonia. New guidelines of the Spanish Society of Pulmonary and Thoracic Surgery (SEPAR) Arch Bronconeumol 2010 46 543 558 20832928 10.1016/j.arbres.2010.06.014

[B27] Levy MM Fink MP Marshall JC Abraham E Angus D Cook D Cohen J Opal SM Vincent JL Ramsay G SCCM/ESICM/ACCP/ATS/SIS SCCM/ESICM/ATS/SIS International Sepsis Definitions Conference Crit Care Med 2003 31 1250 1256 12682500 10.1097/01.CCM.0000050454.01978.3B

[B28] Menéndez R Torres A Zalacaín R Aspa J Martín Villasclaras JJ Borderías L Benítez Moya JM Ruiz-Manzano J Rodríguez de Castro F Blanquer J Pérez D Puzo C Sánchez Gascón F Gallardo J Alvarez C Molinos L Neumofail Group Risk factors of treatment failure in community-acquired pneumonia: implications for disease outcome Thorax 2004 59 960 965 15516472 10.1136/thx.2003.017756 PMC1746855

[B29] Kaplan V Angus DC Griffin MF Clermont G Watson RS Linde-Zwirble WT Hospitalized community-acquired pneumonia in the elderly: age and sex-related patterns of care and outcome in the United States Am J Respir Crit Care Med 2002 165 766 772 11897642 10.1164/ajrccm.165.6.2103038

[B30] Schroder J Kahlke V Staubach KH Zabel P Stuber F Gender differences in human sepsis Arch Surg 1998 133 1200 1205 9820351 10.1001/archsurg.133.11.1200

[B31] Falagas ME Mourtzoukou EG Vardakas KZ Sex differences in the incidence and severity of respiratory tract infections Respir Med 2007 101 1845 1863 17544265 10.1016/j.rmed.2007.04.011

[B32] Warren JL Bacon E Harris T McBean AM Foley DJ Phillips C The burden and outcomes associated with dehydration among US elderly, 1991 Am J Public Health 1994 84 1265 1269 8059883 10.2105/ajph.84.8.1265 PMC1615468

[B33] Bordón J Peyrani P Brock GN Blasi F Rello J File T Ramirez J CAPO Study Group The presence of pneumococcal bacteremia does not influence clinical outcomes in patients with community-acquired pneumonia. Result from the Community-Acquired Pneumonia Organization (CAPO) international cohort study Chest 2008 133 3 618 624 18198264 10.1378/chest.07-1322

[B34] Lisboa T Blot S Waterer GW Canalis E de Mendoza D Rodriguez A Rello J Community-Acquired Pneumonia Intensive Care Units Study Investigators. Radiologic progression of pulmonary infiltrates predicts a worse prognosis in severe community-acquired pneumonia than bacteremia Chest 2009 135 165 172 18689575 10.1378/chest.08-1216

[B35] Martínez JA Horcajada JP Almela M Marco F Soriano A García E Marco MA Torres A Mensa J Addition of a macrolide to a B-lactam-based empirical antibiotic regimen is associated with lower in-hospital mortality for patients with bacteremic pneumococcal pneumonia Clin Infect Dis 2003 36 389 395 12567294 10.1086/367541

[B36] Waterer GW Somes GW Wunderink RG Monotherapy may be suboptimal for severe bacteremic pneumococcal pneumonia Arch Intern Med 2001 161 1837 1842 11493124 10.1001/archinte.161.15.1837

[B37] File TM Mandell LA What is optimal antimicrobial therapy for bacteremic pneumococcal pneumonia? Clin Infect Dis 2003 36 396 398 12567295 10.1086/367545

